# Inner tegument protein pUL37 of herpes simplex virus type 1 is involved in directing capsids to the *trans*-Golgi network for envelopment

**DOI:** 10.1099/vir.0.022053-0

**Published:** 2010-09

**Authors:** David Pasdeloup, Frauke Beilstein, Ashley P. E. Roberts, Marion McElwee, David McNab, Frazer J. Rixon

**Affiliations:** MRC Virology Unit, Institute of Virology, University of Glasgow, Church Street, Glasgow G11 5JR, UK

## Abstract

Secondary envelopment of herpes simplex virus type 1 has been demonstrated as taking place at the *trans*-Golgi network (TGN). The inner tegument proteins pUL36 and pUL37 and the envelope glycoproteins gD and gE are known to be important for secondary envelopment. We compared the cellular localizations of capsids from a virus mutant lacking the UL37 gene with those of a virus mutant lacking the genes encoding gD and gE. Although wild-type capsids accumulated at the TGN, capsids of the pUL37^−^ mutant were distributed throughout the cytoplasm and showed no association with TGN-derived vesicles. This was in contrast to capsids from a gD^−^gE^−^ mutant, which accumulated in the vicinity of TGN vesicles, but did not colocalize with them, suggesting that they were transported to the TGN but were unable to undergo envelopment. We conclude that the inner tegument protein pUL37 is required for directing capsids to the TGN, where secondary envelopment occurs.

Morphogenesis of the herpesvirus particle begins when the capsid is formed in the nucleus of an infected cell (Homa & Brown, 1997). The capsid exits the nucleus by budding at the inner nuclear membrane, thus gaining a primary envelope, which then fuses with the outer nuclear membrane, releasing the capsid into the cytosol (Mettenleiter *et al.*, 2006). How the capsid then acquires the bulk of the tegument and undergoes secondary envelopment is not clear. The *trans*-Golgi network (TGN) appears to be an important site of envelopment where glycoproteins and tegument proteins accumulate (Sugimoto *et al.*, 2008; Turcotte *et al.*, 2005). The fact that TGN targeting of these proteins can occur independently of the presence of capsids has been shown directly for several glycoproteins (Turcotte *et al.*, 2005) and is supported by the observation that L-particles, consisting of enveloped tegument, are formed under conditions where capsid formation is blocked (Rixon *et al.*, 1992; Roberts *et al.*, 2009). A number of viral proteins have been implicated in the secondary envelopment process of herpes simplex virus type 1 (HSV-1) or pseudorabies virus, including the glycoproteins gD, gE, gI and gM (Brack *et al.*, 1999; Farnsworth *et al.*, 2003), the envelope protein pUL20 (Baines *et al.*, 1991; Foster *et al.*, 2004) and the tegument proteins pUL48 (Mossman *et al.*, 2000), pUL11 (Baines & Roizman, 1992; Leege *et al.*, 2009), pUL36 and pUL37 (Desai, 2000; Desai *et al.*, 2001; Roberts *et al.*, 2009). The inner tegument proteins pUL36 and pUL37 interact with each other and the interaction is conserved across members of the family *Herpesviridae* (Bechtel & Shenk, 2002; Klupp *et al.*, 2002; Mijatov *et al.*, 2007; Rozen *et al.*, 2008; Uetz *et al.*, 2006; Vittone *et al.*, 2005). Both proteins are components of both infectious virions and L-particles (McLauchlan *et al.*, 1994; Szilagyi & Cunningham, 1991). Their presence in L-particles implies that their interaction with outer tegument proteins does not depend on the presence of capsids. This was confirmed by recent studies showing that pUL37 associates with the TGN of infected cells and that this localization depends on the presence of pUL36, but not of capsids (Desai *et al.*, 2008). Furthermore, studies with deletion mutants have shown that pUL36 and pUL37 are mutually co-dependent for incorporation into L-particles, suggesting that they normally occur as a complex (Roberts *et al.*, 2009). pUL36 has been reported to interact with the outer tegument protein pUL48 (Vittone *et al.*, 2005) and to be important for its incorporation in virions (Ko *et al.*, 2009). pUL48, in turn, binds to several other tegument proteins and glycoproteins, including pUL46, pUL49 and gH (Elliott *et al.*, 1995; Gross *et al.*, 2003; Lee *et al.*, 2008; Vittone *et al.*, 2005). pUL36 has also been shown to interact with the minor capsid protein pUL25 (Coller *et al.*, 2007; Pasdeloup *et al.*, 2009), and pUL37 has been reported to interact with pUL46 in a yeast two-hybrid assay (Lee *et al.*, 2008). Both pUL36 and pUL37 have essential roles in virion assembly, and virus mutants deleted for either gene accumulate large numbers of unenveloped capsids in the cytoplasm of infected cells (Desai, 2000; Desai *et al.*, 2001; Fuchs *et al.*, 2004; Klupp *et al.*, 2001; Roberts *et al.*, 2009).

Taken together, these observations identify pUL36 and pUL37 as likely candidates to act as a bridge between the capsid and the outer tegument and envelope compartments during virion assembly. Therefore, we compared the behaviour of a UL37-null mutant with that of a gD-/gE-null virus to examine the role of these proteins in targeting capsids to the TGN.

As we wished to observe the behaviour of intracellular capsids and to avoid the poor antibody labelling typically seen with wild-type (WT) HSV-1 capsids, we generated a number of viruses encoding a fluorescently tagged capsid protein. In vVP26GFP, the green fluorescent protein (GFP) fused to the N terminus of the small capsid protein VP26 (Desai & Person, 1998) was recombined into the WT HSV-1 (strain 17+) genome. vVP26GFP was then used to generate the UL37^−^ virus vFRΔ37-VP26GFP by co-infecting the pUL37-expressing cell line 80C02 with FRΔUL37 (Roberts *et al.*, 2009) and vVP26GFP and selecting plaques exhibiting both GFP fluorescence and a defect in growth on non-complementing rabbit skin (RS) cells.

To screen vFRΔ37-VP26GFP for fusion of GFP to VP26 and for the absence of pUL37, RS cells were infected for 24 h with WT HSV-1, vVP26GFP or vFRΔ37-VP26GFP. The cells were then harvested and analysed by Western blot analysis using GFP-, VP26- and UL37-specific antibodies [see Supplementary Methods (available in JGV Online) for antibody details]. Fig. 1(a) shows that pUL37 is present in WT HSV-1-infected and vVP26GFP-infected cells (lanes 4 and 3, respectively), but is missing from vFRΔ37-VP26GFP-infected cells (lane 2). The VP26-specific antibody recognizes a band of 14 kDa in WT HSV-1-infected cells (lane 4, right), but this band is missing in vFRΔ37-VP26GFP-infected and vVP26GFP-infected cells (lanes 2 and 3, right), where a band of approximately 40–45 kDa is recognized by both VP26- and GFP-specific antibodies. This band is of the approximate size expected for the GFP–VP26 fusion protein (39 kDa).

To compare the growth characteristics of vFRΔ37-VP26GFP, vVP26GFP and WT HSV-1, virus stocks were titrated on complementing and non-complementing cells. This confirmed that the Δ37 mutation was lethal for virus growth, with a reduction in titre of >10^5^ (Fig. 1c). Single-step growth-curve analysis on the complementing cell line showed that vFRΔ37-VP26GFP grew with similar kinetics to WT HSV-1 and vVP26GFP, but reached a slightly lower titre (around 7 % lower) after 24 h (see Supplementary Fig. S1, available in JGV Online).

The combined absence of glycoproteins gD and gE prevents virus budding and results in accumulation of tegumented, unenveloped capsids in the cytoplasm (Farnsworth *et al.*, 2003). In order to compare this well-characterized phenotype with that observed with vFRΔ37-VP26GFP, we used vgD-gE-VP26RFP, which contains both a red fluorescent protein (RFP)-tagged VP26 and a gD–gE deletion. The vgD-gE-VP26RFP virus was obtained by co-infecting the gD-expressing cell line VD60 with vUL35RFP1D1, a virus containing an RFP-tagged VP26, and the gD^−^, gE^−^ virus vRR1097-gE*β* (Farnsworth *et al.*, 2003) (see Supplementary Methods for details of virus constructions).

We first characterized these viruses with regard to their growth defect. Titration of virus stocks on complementing (VD60) and non-complementing (Vero) cells confirmed that the deletion of the US6 open reading frame encoding gD was lethal for virus growth, with a reduction in titre of ≥10^5^ for vgD-gE-VP26RFP (Fig. 1c). The higher background level of vgD-VP26RFP seen on the non-complementing cell line was a result of recombination between the mutant genome and the extensive virus sequences used to make the complementing cell line (Ligas & Johnson, 1988). The protein-expression phenotypes of the virus mutants were examined by infecting non-complementing Vero cells with 5 p.f.u. of WT HSV-1, vRR1097-gE*β*, vUL35RFP1D1, vgD-VP26RFP or vgD-gE-VP26RFP per cell and by analysing cell lysates by Western blotting using antibodies against VP26, gD and gE (Fig. 1b). Glycoproteins gD and gE were detected as a pattern of bands, reflecting different states of glycosylation and maturation of the proteins (Cohen *et al.*, 1978). These bands were present in WT HSV-1- and vUL35RFP1D1-infected cells (lanes 1 and 3) and absent from vRR1097-gE*β*- and vgD-gE-VP26RFP-infected cells (lanes 2 and 5). As expected, the vgD-VP26RFP-infected cell lysate showed the presence of gE and the absence of gD (lane 4). The fusion of RFP to VP26 was confirmed by the shift in molecular mass of VP26 from approximately 14 kDa in WT HSV-1- and vRR1097-gE*β***-**infected cells (lanes 1 and 2) to approximately 40 kDa in vUL35RFP1D1-, vgD-VP26RFP- and vgD-gE-VP26RFP-infected cells (lanes 3–5). This experiment confirmed the absence of gD and gE and the fusion of RFP to VP26 in the newly engineered vgD-gE-VP26RFP virus.

To examine the effect of the UL37 and gD–gE mutations on capsid association with the TGN, HFFF_2_ (human fetal foreskin fibroblasts; European Collection of Cell Cultures) and HeLa cells were infected for 15, 18 or 24 h with 5 p.f.u. of vVP26GFP, vFRΔ37-VP26GFP, vUL35RFP1D1 or vgD-gE-VP26RFP per cell, and stained for the TGN using the anti-TGN46 antibody (Fig. 2) or for the Golgi with the anti-giantin antibody (see Supplementary Fig. S2, available in JGV Online). Similar results were observed at all times post-infection and only the 15 h images are shown. The TGN is disrupted into small vesicles upon infection by HSV-1, as described previously (Campadelli *et al.*, 1993). In all cases, the patterns of association between capsids and TGN vesicles were the same in HFFF_2_ and HeLa cells. However, while cytoplasmic capsids formed aggregates in vFRΔ37-VP26GFP-infected HFFF2 cells, as described previously for other UL37^−^ mutants (Desai *et al.*, 2001; Klupp *et al.*, 2001; Roberts *et al.*, 2009), aggregates were not seen in HeLa cells, where their absence made it easier to observe the behaviour of individual capsids. Many of the capsids in the control vVP26GFP- and vUL35RFP1D1-infected cells colocalized with TGN vesicles, although they were also present in other regions of the cytoplasm (Fig. 2a, b). In the case of vgD-gE-VP26RFP, capsids accumulated in clusters adjacent to, but separated from, TGN vesicles (Fig. 2b). The tendency of capsids to aggregate in the absence of gD and gE was described previously for the parental mutant, vRR1097-gE*β* (Farnsworth *et al.*, 2003). In contrast to the juxtaposition of capsids and TGN seen with vgD-gE-VP26RFP, vFRΔ37-VP26GFP capsids accumulated throughout the cytoplasm of infected cells, without exhibiting any association with TGN46- or giantin-positive vesicles (Fig. 2a; Supplementary Fig. S2). The differing behaviours of vFRΔ37-VP26GFP and vgD-gE-VP26RFP were not due to capsid aggregation, as vFRΔ37-VP26GFP capsids also failed to associate with TGN in HFFF_2_ cells, where large aggregates formed readily (Fig. 2d). Quantification of the fluorescence signals confirmed that there was a significant decrease (approx. 90 %) in the level of colocalization of vFRΔ37-VP26GFP and vgD-gE-VP26RFP capsids with TGN, compared with their corresponding controls (Fig. 2c). To confirm that the fluorescent protein tags on VP26 were not influencing the behaviour of capsids, the experiments were repeated using the original untagged versions of the mutants, vFRΔUL37 and vRR1097-gE*β* (see Supplementary Fig. S3, available in JGV Online). In agreement with previous results, WT capsids were largely colocalized with TGN vesicles and vRR1097-gE*β* capsids were aggregated and juxtaposed to TGN vesicles, whereas vFRΔUL37 capsids were widely dispersed and showed no association with the TGN.

As lack of both the inner tegument protein pUL37 and the envelope glycoproteins gD–gE blocked the colocalization of capsids with the TGN, it was important to show that these two classes of structural protein were functioning independently. Immunofluorescence using the anti-UL36 or anti-pUL37 antibody revealed extensive colocalization between pUL36 or pUL37 and vgD-gE-VP26RFP capsid clusters (Fig. 3a). The presence of pUL36 and UL37 implies that the failure of capsids to gain an envelope was a direct result of the absence of the glycoproteins and not due to a block on the association between these capsids and inner tegument proteins. Moreover, we could exclude the possibility that the phenotype observed in vFRΔ37-VP26GFP-infected cells was due to a possible absence of pUL36 on capsids, as vFRΔ37-VP26GFP capsids were stained for pUL36 using the anti-UL36 antibody (Fig. 3b). To examine whether localization of viral glycoproteins to the TGN was affected by the lack of pUL37, vVP26GFP- and vFRΔ37-VP26GFP-infected cells were stained for TGN46 and the glycoprotein gD. In both cases, gD was found on all TGN vesicles (Fig. 3c), either in association with capsids (vVP26GFP-infected cells) or in their absence (vFRΔ37-VP26GFP-infected cells). Similar observations were made with gI and gE (data not shown), demonstrating that pUL37 is not required to direct any of these glycoproteins to the TGN. Similarly, examination of the outer tegument protein pUL48 showed its presence at the TGN in both vVP26GFP- and vFRΔ37-VP26GFP-infected cells (Fig. 3d). Thus, the absence of pUL37 appears to block envelopment by directly preventing the targeting of capsids to the TGN, rather than by interfering with the localization of envelopment-related glycoproteins or outer tegument proteins. It is clear, therefore, that the maturation defects observed in the inner tegument protein and glycoprotein mutants are different in nature, with pUL37 being required to direct capsids to the TGN, and gD and gE being needed for their envelopment once they have arrived there.

The mechanism by which pUL37 helps guide capsids to the TGN is unclear. Directed movement would require the involvement of cellular factors, which might be expected to be recruited to capsids by interacting with pUL37 or with its inner tegument partner, pUL36. However, any such factors remain to be identified. Although our data support a role for pUL37 in directing capsids to the TGN, this does not preclude involvement in later stages of envelopment.

## Supplementary Material

[Supplementary methods, references and figures]

## Figures and Tables

**Fig. 1.**
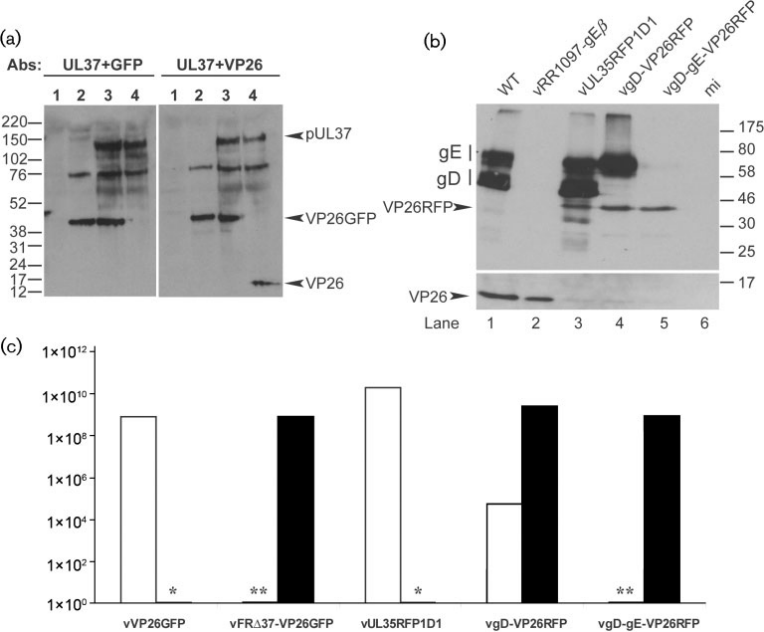
Characterization of the vFRΔ37-VP26GFP and vgD-gE-VP26RFP viruses. (a) RS cells were mock-infected (lane 1) or infected with 5 p.f.u. of vFRΔ37-VP26GFP (lane 2), vVP26GFP (lane 3) or WT HSV-1 (lane 4) per cell and harvested after 24 h. Proteins were analysed by Western blotting using the pUL37 antibody, together with either the GFP-specific antibody (left panel) or the VP26-specific antibody (right panel). Note that a viral protein migrating at approximately 75 kDa is recognized non-specifically by the pUL37 antibody. Molecular mass markers (in kDa) are indicated to the left of the figure. (b) Vero cells were infected with 5 p.f.u. of WT HSV-1 (lane 1), vRR1097-gE*β* (lane 2), vUL35RFP1D1 (lane 3), vgD-VP26RFP (lane 4) or vgD-gE-VP26RFP (lane 5) per cell or were mock-infected (lane 6), and harvested after 24 h. Proteins were analysed by Western blotting using a gD antibody, a gE antibody and a VP26-specific antibody. Molecular mass markers (in kDa) are indicated to the right of the figure. (c) Growth of viruses on complementing (filled bars) and non-complementing (empty bars) cell lines. Concentrated stocks of virus were titrated on RS cells (vVP26GFP), Vero cells (vUL35RFP1D1, vgD-VP26RFP, vgD-gE-VP26RFP), 80C02 cells [a clone of RS cells expressing UL37 (Roberts *et al.*, 2009)] (vFRΔ37-VP26GFP) or VD60 cells [a clone of Vero cells expressing gD (Ligas & Johnson, 1988)] (vgD-VP26RFP, vgD-gE-VP26RFP). *vVP26GFP and vUL35RFP1D1 were not tested on complementing cells. **The titres of vFRΔ37-VP26GFP and vgD-gE-VP26RFP on non-complementing cells were assigned as <10^3^ because the input virus caused severe cytopathic effects at lower dilutions.

**Fig. 2.**
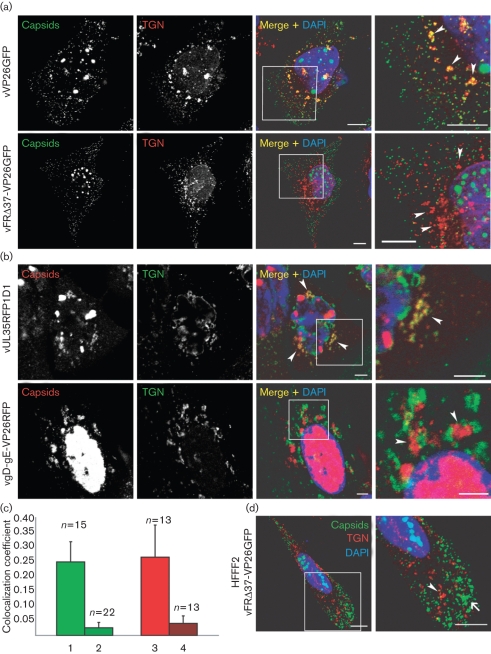
Association of capsids with the TGN. (a) HeLa cells were infected with 5 p.f.u. of vVP26GFP or vFRΔ37-VP26GFP per cell. At 15 h post-infection, the cells were fixed and stained with anti-TGN46 antibody and a GAR_568_ antibody (red). Capsids were visualized through direct GFP fluorescence (green). Bar, 10 μm. (b) HeLa cells were infected with 5 p.f.u. of vUL35RFP1D1 or vgD-gE-VP26RFP per cell. At 15 h post-infection, cells were fixed and labelled with anti-TGN46 antibody and a GAR_Cy5_ antibody (pseudo-coloured in green). Capsids were visualized through direct RFP fluorescence (red). In all cases, nuclei were stained with DAPI (blue). The boxed regions in the Merge+DAPI images are shown enlarged in the final panel and the positions of some TGN-derived vesicles are indicated by arrowheads. (c) Quantification of the amount of TGN signal that colocalizes with capsid signal for the four different viruses in HeLa cells (1, vVP26GFP; 2, vFRΔ37-VP26GFP; 3, vUL35RFP1D1; 4, vgD-gE-VP26RFP). The numbers of fields of view analysed are indicated above each bar. (d) HFFF_2_ cells were infected with 5 p.f.u. of vFRΔ37-VP26GFP per cell and labelled as above. A capsid aggregate is indicated by an arrow and a TGN vesicle by an arrowhead. Bars, 5 μm.

**Fig. 3.**
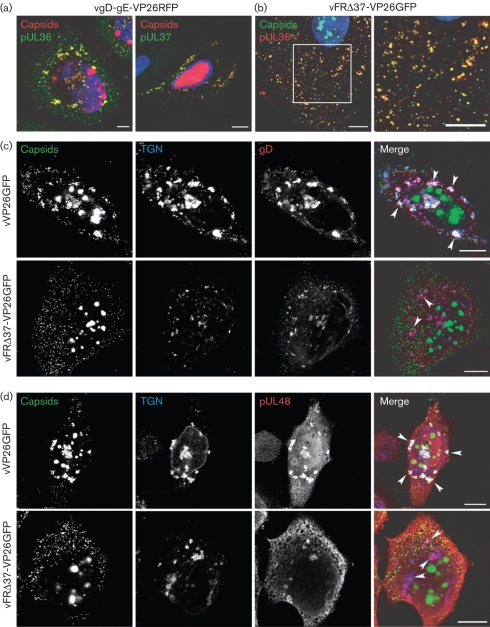
Effect of UL37 and gD–gE deletions on the localization of glycoproteins and tegument proteins. (a) HeLa cells infected with vgD-gE-VP26RFP for 15 h were fixed and labelled with the anti-pUL36 antibody and a GAM_633_ antibody (pseudo-coloured in green) or were permeabilized with digitonin and labelled with anti-pUL37 antibody and a GAR_Cy5_ antibody (pseudo-coloured in green) under native conditions as described by Copeland *et al.* (2009). Bar, 5 μm. (b) HeLa cells were infected with vFRΔ37-VP26GFP for 15 h, fixed and labelled with anti-pUL36 antibody and a GAR_568_ antibody (red). (c) HeLa cells were infected with 5 p.f.u. of vVP26GFP or vFRΔ37-VP26GFP per cell. At 15 h post-infection, cells were fixed and labelled with TGN46-specific antibody and a GAR_Cy5_ antibody to label the TGN (blue), and with a gD-specific antibody and a GAM_568_ antibody to label gD (red). Capsids were visualized through direct GFP fluorescence (green). (d) Cells were infected and treated as in (c) except that the gD-specific antibody was replaced by pUL48-specific antibody to label pUL48 (red). Arrowheads in (c) and (d) indicate the positions of some TGN-derived vesicles. Bars, 10 μm.

## References

[r1] Baines, J. D. & Roizman, B. (1992). The UL11 gene of herpes simplex virus 1 encodes a function that facilitates nucleocapsid envelopment and egress from cells. J Virol 66, 5168–5174. 1321297 10.1128/jvi.66.8.5168-5174.1992 PMC241400

[r2] Baines, J. D., Ward, P. L., Campadelli-Fiume, G. & Roizman, B. (1991). The UL20 gene of herpes simplex virus 1 encodes a function necessary for viral egress. J Virol 65, 6414–6424. 1719228 10.1128/jvi.65.12.6414-6424.1991 PMC250678

[r3] Bechtel, J. T. & Shenk, T. (2002). Human cytomegalovirus UL47 tegument protein functions after entry and before immediate-early gene expression. J Virol 76, 1043–1050. 11773380 10.1128/JVI.76.3.1043-1050.2002 PMC135821

[r4] Brack, A. R., Dijkstra, J. M., Granzow, H., Klupp, B. G. & Mettenleiter, T. C. (1999). Inhibition of virion maturation by simultaneous deletion of glycoproteins E, I, and M of pseudorabies virus. J Virol 73, 5364–5372. 10364283 10.1128/jvi.73.7.5364-5372.1999 PMC112592

[r5] Campadelli, G., Brandimarti, R., Di Lazzaro, C., Ward, P. L., Roizman, B. & Torrisi, M. R. (1993). Fragmentation and dispersal of Golgi proteins and redistribution of glycoproteins and glycolipids processed through the Golgi apparatus after infection with herpes simplex virus 1. Proc Natl Acad Sci U S A 90, 2798–2802. 8385343 10.1073/pnas.90.7.2798 PMC46183

[r6] Cohen, G. H., Katze, M., Hydrean-Stern, C. & Eisenberg, R. J. (1978). Type-common CP-1 antigen of herpes simplex virus is associated with a 59,000-molecular-weight envelope glycoprotein. J Virol 27, 172–181. 80458 10.1128/jvi.27.1.172-181.1978 PMC354150

[r7] Coller, K. E., Lee, J. I., Ueda, A. & Smith, G. A. (2007). The capsid and tegument of the alphaherpesviruses are linked by an interaction between the UL25 and VP1/2 proteins. J Virol 81, 11790–11797. 17715218 10.1128/JVI.01113-07 PMC2168758

[r8] Copeland, A. M., Newcomb, W. W. & Brown, J. C. (2009). Herpes simplex virus replication: roles of viral proteins and nucleoporins in capsid-nucleus attachment. J Virol 83, 1660–1668. 19073727 10.1128/JVI.01139-08 PMC2643781

[r9] Desai, P. J. (2000). A null mutation in the UL36 gene of herpes simplex virus type 1 results in accumulation of unenveloped DNA-filled capsids in the cytoplasm of infected cells. J Virol 74, 11608–11618. 11090159 10.1128/jvi.74.24.11608-11618.2000 PMC112442

[r10] Desai, P. & Person, S. (1998). Incorporation of the green fluorescent protein into the herpes simplex virus type 1 capsid. J Virol 72, 7563–7568. 9696854 10.1128/jvi.72.9.7563-7568.1998 PMC110002

[r11] Desai, P., Sexton, G. L., McCaffery, J. M. & Person, S. (2001). A null mutation in the gene encoding the herpes simplex virus type 1 UL37 polypeptide abrogates virus maturation. J Virol 75, 10259–10271. 11581394 10.1128/JVI.75.21.10259-10271.2001 PMC114600

[r12] Desai, P., Sexton, G. L., Huang, E. & Person, S. (2008). Localization of herpes simplex virus type 1 UL37 in the Golgi complex requires UL36 but not capsid structures. J Virol 82, 11354–11361. 18787001 10.1128/JVI.00956-08 PMC2573249

[r13] Elliott, G., Mouzakitis, G. & O'Hare, P. (1995). VP16 interacts via its activation domain with VP22, a tegument protein of herpes simplex virus, and is relocated to a novel macromolecular assembly in coexpressing cells. J Virol 69, 7932–7941. 7494306 10.1128/jvi.69.12.7932-7941.1995 PMC189738

[r14] Farnsworth, A., Goldsmith, K. & Johnson, D. C. (2003). Herpes simplex virus glycoproteins gD and gE/gI serve essential but redundant functions during acquisition of the virion envelope in the cytoplasm. J Virol 77, 8481–8494. 12857917 10.1128/JVI.77.15.8481-8494.2003 PMC165244

[r15] Foster, T. P., Melancon, J. M., Baines, J. D. & Kousoulas, K. G. (2004). The herpes simplex virus type 1 UL20 protein modulates membrane fusion events during cytoplasmic virion morphogenesis and virus-induced cell fusion. J Virol 78, 5347–5357. 15113914 10.1128/JVI.78.10.5347-5357.2004 PMC400383

[r16] Fuchs, W., Klupp, B. G., Granzow, H. & Mettenleiter, T. C. (2004). Essential function of the pseudorabies virus UL36 gene product is independent of its interaction with the UL37 protein. J Virol 78, 11879–11889. 15479829 10.1128/JVI.78.21.11879-11889.2004 PMC523282

[r17] Gross, S. T., Harley, C. A. & Wilson, D. W. (2003). The cytoplasmic tail of herpes simplex virus glycoprotein H binds to the tegument protein VP16 *in vitro* and *in vivo*. Virology 317, 1–12. 14675620 10.1016/j.virol.2003.08.023

[r18] Homa, F. L. & Brown, J. C. (1997). Capsid assembly and DNA packaging in herpes simplex virus. Rev Med Virol 7, 107–122. 10398476 10.1002/(sici)1099-1654(199707)7:2<107::aid-rmv191>3.0.co;2-m

[r19] Klupp, B. G., Granzow, H., Mundt, E. & Mettenleiter, T. C. (2001). Pseudorabies virus UL37 gene product is involved in secondary envelopment. J Virol 75, 8927–8936. 11533156 10.1128/JVI.75.19.8927-8936.2001 PMC114461

[r20] Klupp, B. G., Fuchs, W., Granzow, H., Nixdorf, R. & Mettenleiter, T. C. (2002). Pseudorabies virus UL36 tegument protein physically interacts with the UL37 protein. J Virol 76, 3065–3071. 11861875 10.1128/JVI.76.6.3065-3071.2002 PMC135998

[r21] Ko, D. H., Cunningham, A. L. & Diefenbach, R. J. (2009). The major determinant for addition of tegument protein pUL48 (VP16) to capsids in herpes simplex virus type 1 is the presence of the major tegument protein pUL36 (VP1/2). J Virol 84, 1397–1405. 19923173 10.1128/JVI.01721-09 PMC2812353

[r22] Lee, J. H., Vittone, V., Diefenbach, E., Cunningham, A. L. & Diefenbach, R. J. (2008). Identification of structural protein–protein interactions of herpes simplex virus type 1. Virology 378, 347–354. 18602131 10.1016/j.virol.2008.05.035

[r23] Leege, T., Fuchs, W., Granzow, H., Kopp, M., Klupp, B. G. & Mettenleiter, T. C. (2009). Effects of simultaneous deletion of pUL11 and glycoprotein M on virion maturation of herpes simplex virus type 1. J Virol 83, 896–907. 19004941 10.1128/JVI.01842-08 PMC2612385

[r24] Ligas, M. W. & Johnson, D. C. (1988). A herpes simplex virus mutant in which glycoprotein D sequences are replaced by *β*-galactosidase sequences binds to but is unable to penetrate into cells. J Virol 62, 1486–1494. 2833603 10.1128/jvi.62.5.1486-1494.1988 PMC253172

[r25] McLauchlan, J., Liefkens, K. & Stow, N. D. (1994). The herpes simplex virus type 1 UL37 gene product is a component of virus particles. J Gen Virol 75, 2047–2052. 8046407 10.1099/0022-1317-75-8-2047

[r26] Mettenleiter, T. C., Klupp, B. G. & Granzow, H. (2006). Herpesvirus assembly: a tale of two membranes. Curr Opin Microbiol 9, 423–429. 16814597 10.1016/j.mib.2006.06.013

[r27] Mijatov, B., Cunningham, A. L. & Diefenbach, R. J. (2007). Residues F593 and E596 of HSV-1 tegument protein pUL36 (VP1/2) mediate binding of tegument protein pUL37. Virology 368, 26–31. 17651773 10.1016/j.virol.2007.07.005

[r28] Mossman, K. L., Sherburne, R., Lavery, C., Duncan, J. & Smiley, J. R. (2000). Evidence that herpes simplex virus VP16 is required for viral egress downstream of the initial envelopment event. J Virol 74, 6287–6299. 10864638 10.1128/jvi.74.14.6287-6299.2000 PMC112134

[r29] Pasdeloup, D., Blondel, D., Isidro, A. L. & Rixon, F. J. (2009). Herpesvirus capsid association with the nuclear pore complex and viral DNA release involve the nucleoporin CAN/Nup214 and the capsid protein pUL25. J Virol 83, 6610–6623. 19386703 10.1128/JVI.02655-08 PMC2698519

[r30] Rixon, F. J., Addison, C. & McLauchlan, J. (1992). Assembly of enveloped tegument structures (L particles) can occur independently of virion maturation in herpes simplex virus type 1-infected cells. J Gen Virol 73, 277–284. 1311357 10.1099/0022-1317-73-2-277

[r31] Roberts, A. P., Abaitua, F., O'Hare, P., McNab, D., Rixon, F. J. & Pasdeloup, D. (2009). Differing roles of inner tegument proteins pUL36 and pUL37 during entry of herpes simplex virus type 1. J Virol 83, 105–116. 18971278 10.1128/JVI.01032-08 PMC2612316

[r32] Rozen, R., Sathish, N., Li, Y. & Yuan, Y. (2008). Virion-wide protein interactions of Kaposi's sarcoma-associated herpesvirus. J Virol 82, 4742–4750. 18321973 10.1128/JVI.02745-07 PMC2346726

[r33] Sugimoto, K., Uema, M., Sagara, H., Tanaka, M., Sata, T., Hashimoto, Y. & Kawaguchi, Y. (2008). Simultaneous tracking of capsid, tegument, and envelope protein localization in living cells infected with triply fluorescent herpes simplex virus 1. J Virol 82, 5198–5211. 18353954 10.1128/JVI.02681-07 PMC2395178

[r34] Szilagyi, J. F. & Cunningham, C. (1991). Identification and characterization of a novel non-infectious herpes simplex virus-related particle. J Gen Virol 72, 661–668. 1848601 10.1099/0022-1317-72-3-661

[r35] Turcotte, S., Letellier, J. & Lippe, R. (2005). Herpes simplex virus type 1 capsids transit by the *trans*-Golgi network, where viral glycoproteins accumulate independently of capsid egress. J Virol 79, 8847–8860. 15994778 10.1128/JVI.79.14.8847-8860.2005 PMC1168770

[r36] Uetz, P., Dong, Y. A., Zeretzke, C., Atzler, C., Baiker, A., Berger, B., Rajagopala, S. V., Roupelieva, M., Rose, D. & other authors (2006). Herpesviral protein networks and their interaction with the human proteome. Science 311, 239–242. 16339411 10.1126/science.1116804

[r37] Vittone, V., Diefenbach, E., Triffett, D., Douglas, M. W., Cunningham, A. L. & Diefenbach, R. J. (2005). Determination of interactions between tegument proteins of herpes simplex virus type 1. J Virol 79, 9566–9571. 16014918 10.1128/JVI.79.15.9566-9571.2005 PMC1181608

