# Supplementary material for: Inner tegument protein pUL37 of herpes simplex virus type 1 is involved in directing capsids to the trans-Golgi network for envelopment
Source: J Gen Virol. 2010 Sep;91(Pt 9):2145–51. doi: 10.1099/vir.0.022053-0 (PMC3066548; doi:10.1099/vir.0.022053-0)
Supplement: [Supplementary methods, references and figures] [file supp_91_9_2145__index.html]

 Inner tegument protein pUL37 of herpes simplex virus type 1 is involved in directing capsids to the trans-Golgi network for envelopment -- Pasdeloup et al. 91 (9): 2145 Data Supplement - Supplementary methods, references and figures -- Journal of General Virology

### Inner tegument protein pUL37 of herpes simplex virus type 1 is involved in directing capsids to the *trans*-Golgi network for envelopment, by D. Pasdeloup, F. Beilstein, A. P. E. Roberts, M. McElwee, D. McNab and F. J. Rixon

*Journal of General Virology* vol. **91**, part 9, pp. 2145–2151

  

**Supplementary Methods** and references [PDF file] (68 KB)

  

**Supplementary Fig. S1.** Single-step growth-curve analysis of vFRΔ37-VP26GFP.

**Supplementary Fig. S2.** Association of capsids with the Golgi in cells infected with vVP26GFP or vFRΔ37-VP26GFP as assessed by giantin labelling.

**Supplementary Fig. S3.** Association of untagged capsids with the TGN. HeLa cells were mock-infected or infected with 5 p.f.u of WT HSV-1, vRR1097-gE*β* or vFRΔUL37.

[Single PDF file of figures] (474 KB)

  
  
