# Supplementary material for: Inner tegument protein pUL37 of herpes simplex virus type 1 is involved in directing capsids to the trans-Golgi network for envelopment
Source: J Gen Virol. 2010 Sep;91(Pt 9):2145–51. doi: 10.1099/vir.0.022053-0 (PMC3066548; doi:10.1099/vir.0.022053-0)
Supplement: [Supplementary methods, references and figures] [file supp_91_9_2145__1.pdf]

Supplementary Methods

Construction of vUL35RFP1D1. vUL35RFP1D1 was obtained by releasing HSV-1 sequences including the UL35 gene from Cos14 (Cunningham & Davison, 1993) as a *Pac*I fragment and ligated into *Pac*I-digested CC6FOS (provided by L. Feldman, University of California Los Angeles, CA, USA) to produce CC6FOS14. To remove the loxP site from the CC6FOS vector, it was replaced by a neomycin cassette flanked by FRT recombination sequences by Red/ET recombination by using a Counter Selection BAC Modification kit (Genebridges). The *neo* sequences were removed by Flp recombinase to generate FOS14ΔLOX. Using the same approach, the neomycin cassette flanked with loxP and variant 2272 recombination sequences (Siegel *et al.*, 2001) was introduced at the N terminus of UL35 to generate Fos14ΔLOXUL35Neo. This was replaced with the pDsRed Monomer sequence (BD Bioscience) by Cre–lox recombination to position the RFP ORF in frame with and upstream of the UL35 ORF. The resulting fosmid was transfected into BHK cells together with WT HSV-1 DNA. Progeny plaques exhibiting red fluorescence were selected, purified through four cycles of plaque picking, grown to high titre and designated vUL35RFP1D1.

Construction of vgD-gE-VP26RFP. vgD-gE-VP26RFP was obtained by co-infecting the gD-expressing cell line VD60 with vUL35RFP1D1 and the gD^{-}, gE^{-} virus vRR1097-gE*β* (Farnsworth *et al.*, 2003). The growth medium was harvested after 24 h and the progeny were serially diluted on VD60 cells. Plaques positive for RFP and also for GFP and *β*-galactosidase [indicating the deletion or disruption of the US6 and US8 ORFs, respectively (Farnsworth *et al.*, 2003)] were isolated and tested for their ability to grow on VD60 and Vero cells. An isolate unable to grow on Vero cells was selected. After three further rounds of purification on VD60 cells, the virus was grown to high titre. An isolate showing RFP and GFP fluorescence, but no *β*-galactosidase signal, was also selected and termed vgD-VP26RFP.

Antibodies. The following primary antibodies were used: anti-GFP (mouse monoclonal GSN24; Sigma), anti-VP26 [1201, raised in rabbit against purified VP26), anti-UL37 (M780, rabbit polyclonal provided by F. Jenkins, University of Pittsburgh, PA, USA (Shelton *et al.*, 1994)], anti-pUL36 [mouse monoclonal #E12-E3; provided by P. O'Hare, Marie Curie Research Institute, Oxted, Surrey, UK (Abaitua & O'Hare, 2008)], anti-VP5 [mAbDM165 (McClelland *et al.*, 2002)], anti-TGN46 (rabbit polyclonal; Sigma), anti-giantin (mouse monoclonal 9B6; Abcam), anti-gI [mAb3104 (Johnson *et al.*, 1988)], anti-gD [mAb4846, (McLauchlan *et al.*, 1994)], anti-gE [mAb3114 (Johnson *et al.*, 1988)] and anti-pUL48 (mAb1-21; SantaCruz Biotechnology).

For immunofluorescence studies, goat or human serum (5 %) was used to block the Fc receptor-binding sites of the gE–gI complex prior to incubation with the antibodies.

Pasdeloup, D., Beilstein, F., Roberts, A. P. E., McElwee, M., McNab, D. & Rixon, F. J. (2010). Inner tegument protein pUL37 of herpes simplex virus type 1 is involved in directing capsids to the *trans*-Golgi network for envelopment. *J Gen Virol* **91**, 2145–2151.

The secondary antibodies used were: Alexa Fluor 488- or 568 conjugated goat anti-mouse (GAM_{488} and GAM_{568}), Alexa Fluor 568 conjugated goat anti-rabbit (GAR_{568}) and Alexa Fluor 633-conjugated goat anti-mouse (GAM_{633}) antibodies (Molecular Probes); Cy5-conjugated goat anti-rabbit (GAR_{Cy5}) antibody (Jackson Immunoresearch); horseradish peroxidase-conjugated goat anti-mouse (GAM_{HRP}) and goat anti-rabbit (GAR_{HRP}) antibodies (Sigma).

Colocalization quantification. Immunofluorescence pictures of single cells were analysed by using the Histogram-Colocalization function of the LSM510 software (version 4; SP2). The colocalization coefficient was evaluated by the software as a percentage of the total fluorescence from one channel (TGN signal) colocalizing with the fluorescence of the second channel (capsid signal) at a fixed intensity threshold of 150 (out of 255) for each channel.

References

Abaitua, F. & O'Hare, P. (2008). Identification of a highly conserved, functional nuclear localization signal within the N-terminal region of herpes simplex virus type 1 VP1–2 tegument protein. *J Virol* **82**, 5234–5244. Medline

Cunningham, C. & Davison, A. J. (1993). A cosmid-based system for constructing mutants of herpes simplex virus type 1. *Virology* **197**, 116–124. Medline

Farnsworth, A., Goldsmith, K. & Johnson, D. C. (2003). Herpes simplex virus glycoproteins gD and gE/gI serve essential but redundant functions during acquisition of the virion envelope in the cytoplasm. *J Virol* **77**, 8481–8494. Medline

Johnson, D. C., Frame, M. C., Ligas, M. W., Cross, A. M. & Stow, N. D. (1988). Herpes simplex virus immunoglobulin G Fc receptor activity depends on a complex of two viral glycoproteins, gE and gI. *J Virol* **62**, 1347–1354. Medline

McClelland, D. A., Aitken, J. D., Bhella, D., McNab, D., Mitchell, J., Kelly, S. M., Price, N. C. & Rixon, F. J. (2002). pH reduction as a trigger for dissociation of herpes simplex virus type 1 scaffolds. *J Virol* **76**, 7407–7417. Medline

McLauchlan, J., Liefkens, K. & Stow, N. D. (1994). The herpes simplex virus type 1 UL37 gene product is a component of virus particles. *J Gen Virol* **75**, 2047–2052. Medline

Shelton, L. S., Albright, A. G., Ruyechan, W. T. & Jenkins, F. J. (1994). Retention of the herpes simplex virus type 1 (HSV-1) UL37 protein on single-stranded DNA columns requires the HSV-1 ICP8 protein. *J Virol* **68**, 521–525. Medline

Siegel, R. W., Jain, R. & Bradbury, A. (2001). Using an in vivo phagemid system to identify non-compatible loxP sequences. *FEBS Lett* **505**, 467–473. Medline

---

Pasdeloup, D., Beilstein, F., Roberts, A. P. E., McElwee, M., McNab, D. & Rixon, F. J. (2010). Inner tegument protein pUL37 of herpes simplex virus type 1 is involved in directing capsids to the *trans*-Golgi network for envelopment. *J Gen Virol* **91**, 2145–2151.
