# Supplementary material for: Inner tegument protein pUL37 of herpes simplex virus type 1 is involved in directing capsids to the trans-Golgi network for envelopment
Source: J Gen Virol. 2010 Sep;91(Pt 9):2145–51. doi: 10.1099/vir.0.022053-0 (PMC3066548; doi:10.1099/vir.0.022053-0)
Supplement: [Supplementary methods, references and figures] [file supp_91_9_2145__2.pdf]

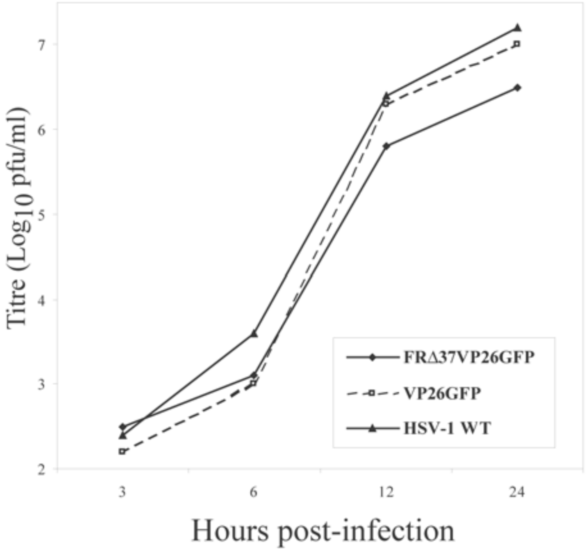

Supplementary Fig. S1. Single-step growth-curve analysis of vFRΔ37-VP26GFP. Replicate 60 mm dishes of cells were infected with 5 p.f.u. of WT HSV-1, vFRΔ37-VP26GFP or vVP26GFP per cell. After 1 h infection, the cells were washed at low pH to remove residual input infectivity, overlaid with 2 ml DMEM and incubation was continued at 37 °C. At 3, 6, 12 and 24 h after infection, cells were harvested by scraping into the growth medium and the progeny virus was titrated. WT HSV-1 and vVP26GFP were grown on RS cells, whereas vFRΔ37-VP26GFP was grown on 80C02 complementing cells.

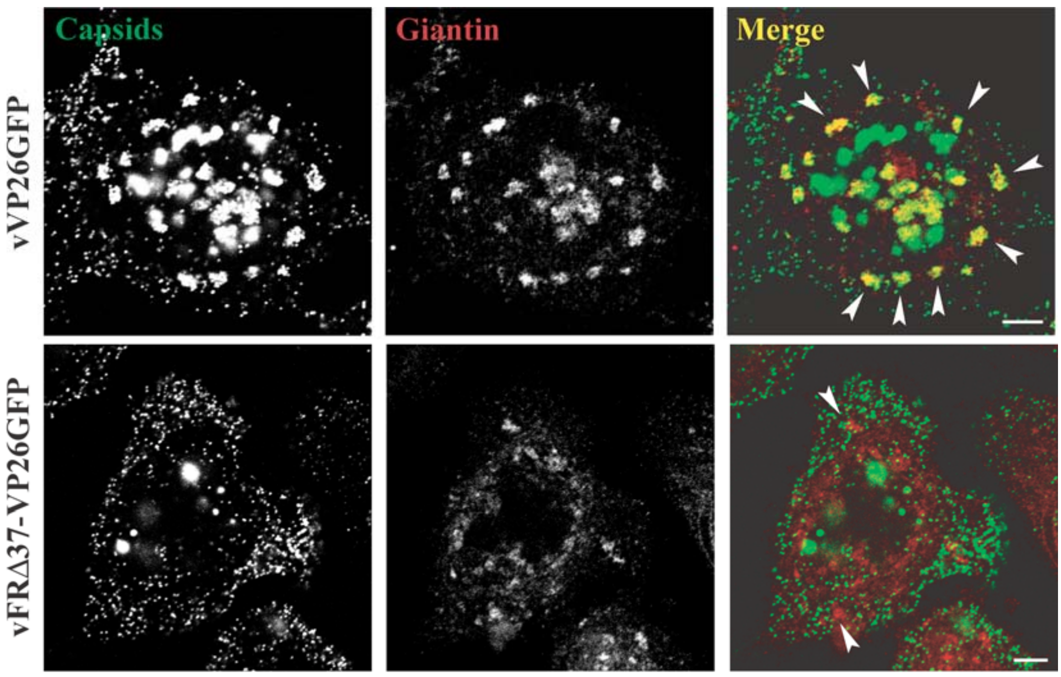

Supplementary Fig. S2. Association of capsids with the Golgi in cells infected with vVP26GFP or vFRΔ37-VP26GFP as assessed by giantin labelling. HeLa cells were infected with 5 p.f.u. of the indicated virus per cell. After 15 h infection, they were fixed and the Golgi was labelled using the monoclonal anti-giantin antibody and a GAM_{568} antibody (red). Arrowheads indicate Golgi-derived vesicles. Bars, 5 µm.

Pasdeloup, D., Beilstein, F., Roberts, A. P. E., McElwee, M., McNab, D. & Rixon, F. J. (2010). Inner tegument protein pUL37 of herpes simplex virus type 1 is involved in directing capsids to the *trans*-Golgi network for envelopment. *J Gen Virol* **91**, 2145–2151.

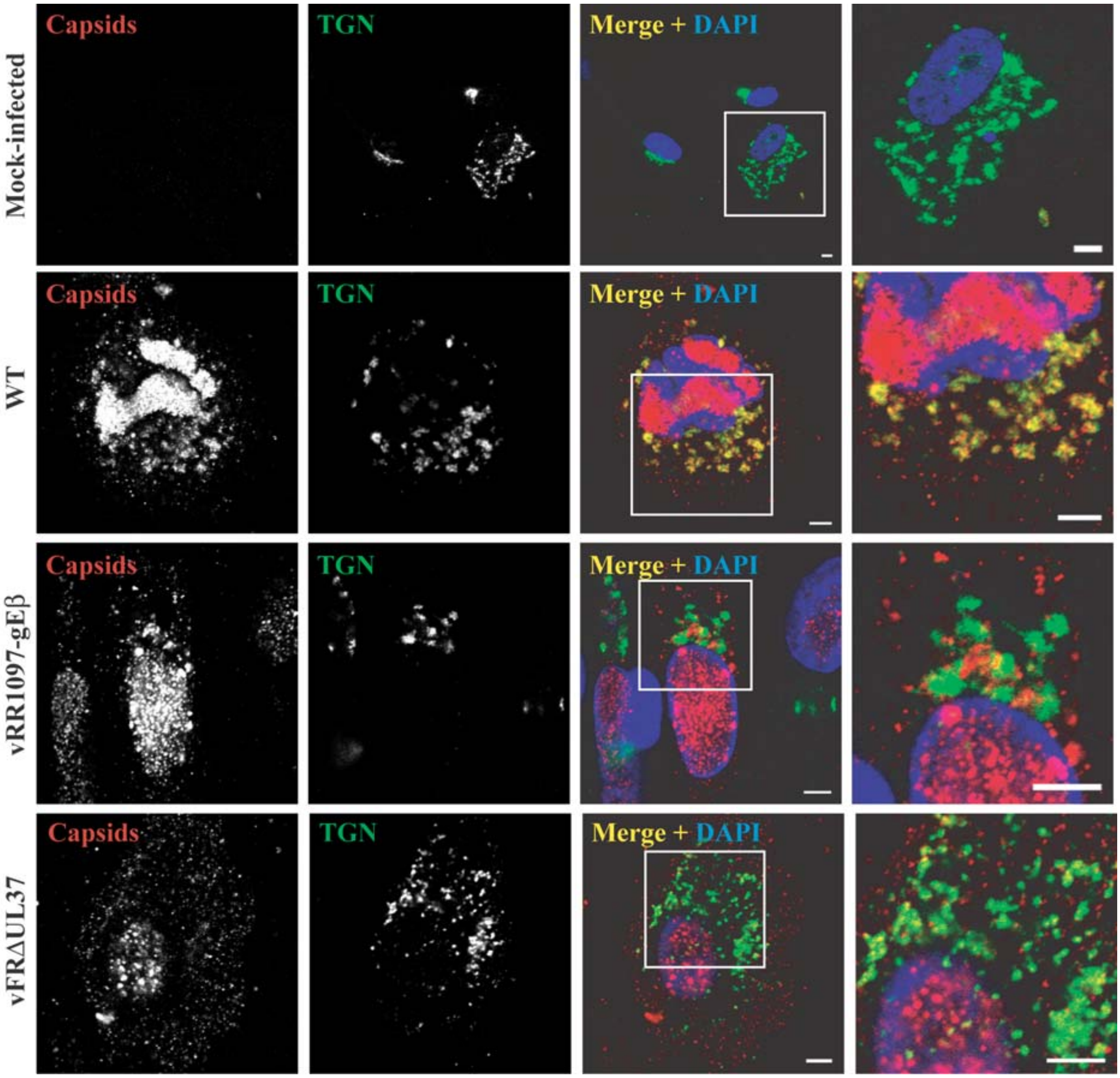

Supplementary Fig. S3. Association of untagged capsids with the TGN. HeLa cells were mock-infected or infected with 5 p.f.u of WT HSV-1, vRR1097-gE*β* or vFRΔUL37. per cell. At 15 h post-infection, they were fixed and stained with the mouse monoclonal DM165 anti-VP5 antibody and GAM_{568} to label capsids (red) and anti-TGN46 antibody and GAR_{488} antibody (green). Bar, 5 µm.

Pasdeloup, D., Beilstein, F., Roberts, A. P. E., McElwee, M., McNab, D. & Rixon, F. J. (2010). Inner tegument protein pUL37 of herpes simplex virus type 1 is involved in directing capsids to the *trans*-Golgi network for envelopment. *J Gen Virol* **91**, 2145–2151.
